# Carbon removal beyond the trees

**DOI:** 10.1038/s43247-025-02226-z

**Published:** 2025-04-02

**Authors:** Emily Cox, Sean Low, Chad M. Baum, Livia Fritz, Laurie Waller, Elspeth Spence, Nick Pidgeon, Rob Bellamy

**Affiliations:** 1 Smith School of Enterprise and the Environment, University of Oxford, Oxford, United Kingdom; 2 Understanding Risk Group, School of Psychology, Cardiff University, Cardiff, Wales; 3 Department of Business Development and Technology, Aarhus University, Herning, Denmark; 4 Wageningen University and Research, Wageningen, Gelderland Netherlands; 5 Department of Geography, University of Manchester, Manchester, United Kingdom

**Keywords:** Psychology, Geography, Carbon and energy

## Abstract

The idea of planting trees to sequester carbon is so popular that it seems to make people feel more negative towards other techniques, when presented with a range of options for carbon removal. Such a bias could hamper development of a broad and socially-robust portfolio of carbon removal options.

Planting trees is the archetype of carbon dioxide removal from the atmosphere. The basic process of photosynthesis is one that people remember from their childhood at school, and the notion of using plants to purify the air dates back to the 18th Century. In a landscape of novel techniques for carbon removal, many of which are unproven at scale, tree planting stands out as simple, intuitive, and already operational at large scale around the world.

However, we also know that meeting climate targets will require much more than planting trees. There is over-reliance on tree planting as a ‘silver bullet’ solution to climate mitigation^1^: current climate mitigation pledges require around 500 million hectares of land – roughly the equivalent of the entire European Union – for tree planting alone^2^. Moreover, trees cannot provide the secure, permanent CO_2_ storage required for mitigating long-lived fossil emissions, and are vulnerable to re-release of CO_2_ by wildfires, disease, and changes in land management. A broad portfolio of methods is therefore essential to hedge against risks and manage trade-offs.

Achieving carbon removal at scale not only requires technologies – it also requires public acceptance and engagement at many interconnected scales, from local communities and directly-affected groups, all the way up to national and supra-national policy formation. Carbon removal will also ultimately be paid for by the general public, through their taxes or purchases. Scaling up carbon removal therefore means appreciating the importance of public opinion.

Here we suggest that the idea of planting trees for carbon removal is so powerful in the public imagination that it could act to bias members of the public away from any other techniques for carbon removal. Multiple projects on public perceptions that we have conducted around the globe have repeatedly suggested such an effect. We argue that the question of how the omnipresence of tree planting in people’s minds influences discussions of carbon removal is in need of empirical, experimental, and qualitative research.

## Messing with nature

Our understanding of how and why perceptions are formed provides some insights as to why planting trees may exert a strong effect. Firstly, tampering with nature has long been shown to be a key predictor of risk perceptions for a wide range of technologies^3^. Perceptions of what is termed naturalness is the strongest factor driving public perceptions of carbon removal^4^. Trees are seen as archetypal symbols of nature, and as providing multiple co-benefits for ecosystems and biodiversity^5,6^. However, it is crucial to emphasise that what is seen as a natural climate solution is not self-evident, because the definitions and boundaries are fuzzy, context-dependent, and socially-constructed^7,8^.

Trees are also familiar, which psychometric theory suggests may result in lower risk perceptions. People may experience a personal connectedness to the notion of forests which does not occur for other carbon removal techniques^6^. However, whether familiarity is a consistent driver of perceptions is debated^4^.

## Trees are always in the frame

In a 2018 project on public perceptions of carbon removal^9^, conducted with randomly-recruited members of the general public in the UK and US, we initially included afforestation as one of four carbon removal techniques for discussion in deliberative workshops, alongside Direct Air Capture and Storage, Bioenergy with Carbon Capture and Storage, and Enhanced Rock Weathering. During piloting, it became clear that the presence of afforestation as an option was making it much more challenging to discuss the other three techniques on their own merits: participants were essentially wondering why we should even be considering these other techniques. We therefore made the decision to drop afforestation from the study due to this biasing effect. We also ran a nationally-representative public survey in the UK and US (*n* = 2026), and found that “trees” was the most common response when asked for the first thought or image that came to mind when they heard the term “carbon dioxide removal”^10^.

In 2023, we ran a separate UK project on perceptions of biological carbon removal techniques, which capture and store carbon using plants, soils and other biological matter. Planting trees is one example of a biological technique, but we were mainly interested in novel biological techniques such as biochar, peatland restoration, and perennial biomass crops. In that study, we tried to avoid the strong framing effect of afforestation by removing all mention from stimulus materials, in a similar way to how researchers have handled other strong framing effects such as that of “naturalness”^11^. Yet the topic was raised unprompted by participants, who in some cases challenged the facilitators for failing to include it as an option: *“Oh I think we’ve touched on it or skirted around it, but it’s always been on the periphery hasn’t it?” [Facilitator: What’s been on the periphery?] “Well, planting more forests, not cutting down the rainforests, all that sort of thing. There are different ways here you can do it naturally without going through huge industrial processes”*^12^.

In other words, we found that it is not possible to remove from participants’ framing something as widely-known and appreciated as trees. Here, planting trees make the other carbon removal methods – including other biological methods – seem more “huge” and “industrial” (in a negative way), when considered in direct comparison to planting forests. A participant in Wales said: *“If there are lots of initiatives that are more about restoration, planting trees, restoring things, I think people are going to be way more on board than destroying and creating”*. The implication here is that planting trees can mitigate the human tendency to “create in order to destroy”, as found similarly in previous work on perceptions of carbon removal^9^.

The topic of co-benefits also tends to come out strongly in support of trees. A participant in Northern Ireland said, *“probably the land could be used for better things: surely there’s something that could be growing there, more trees or something, that would be better. And trees do provide a lot of different resources for people and animals…”* This notion of trees being a ‘better way of using the land’ was also found in previous work on biochar^13^. Trees were also seen as preferable in terms of how quickly they would have an effect, crucial in a context of climate urgency (cf^14^.): *“I think another conclusion we can draw is for carbon capture, plants are involved. To me that means more trees, which is doable now”*.

The underlying question that runs throughout all this is: why would you wish to innovate other carbon removal techniques, when something already exists that can sequester carbon in an effective and allegedly natural way? For many high-emitting countries, this may create a significant discrepancy between public preferences and domestic removal potential^15^.

## Taking it global

**Fig. 1:**
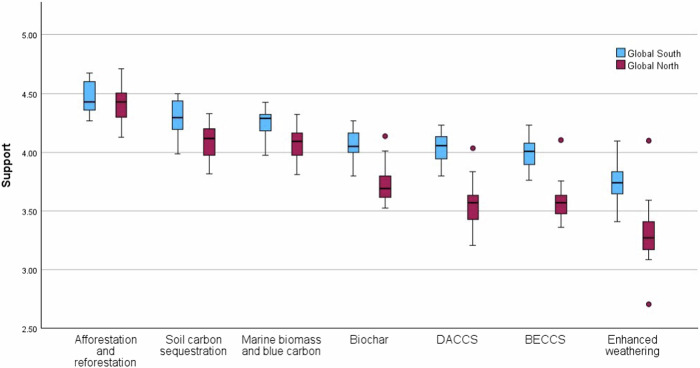
Support for carbon removal methods. Levels of support for different carbon removal methods, from a survey in 30 countries (*n* = 30,284)^16^. Significant differences (*p* < 0.01) between levels of support in Global North versus Global South were identified using nonparametric independent-samples Mann Whitney U testing for six of the technologies: afforestation and reforestation was the exception (*p* = 0.497). Support was assessed on a 1-5 scale (1 = Strictly reject; 3 = Neither support nor reject; 5 = Fully support), along with a “don’t know” option, which was coded as a missing value. Support refers to overall support consisting of three types of activities: research, small-scale field trials, and broad deployment. Boxes show interquartile range and median. Whiskers show either minimum/maximum values in the sample population, or 1.5 times the interquartile range, whichever is closer to the median. Circles show outliers within 1.5 times the IQR.

Another of our projects found similar tendencies around the world. We ran a survey in 30 countries, and focus groups in urban and rural areas in 22 countries, with randomly-recruited members of the public using quotas to ensure socio-demographic representation. The countries chosen represented a roughly even split between Global North and Global South, with every continent represented. Here, afforestation and reforestation was explicitly included, alongside six other carbon removal methods in the survey (see Fig. 1) and four others in the focus groups. Efforts were similarly made to mitigate the well-known naturalism bias, for example by avoiding the un-contextual use of “natural”, and emphasising dynamic socio-environmental interactions in plain language.

Survey participants were significantly more likely to express familiarity with afforestation and reforestation^16^. Compared to evaluations and preferences for most carbon removal options, those for afforestation are more likely to be stable and informed by actual experiences and discussions. Afforestation and reforestation were also the most strongly supported of all the carbon removal options in the survey (Fig. 1) and participants perceived this option to have the most positive balance of benefits to risks. Interestingly, afforestation was the only option without significant differences between Global North and Global South countries, potentially echoing greater familiarity, and indicating broader alignment on perceptions of afforestation around the globe.

The survey did not experimentally test for the impact of including afforestation or reforestation on the perceptions of the other techniques; we refer to the data here solely as an illustration of general baseline preferences for afforestation and reforestation, and the Global South/North comparison. For indications that a biasing effect may occur, we turn to the qualitative data from this study.

Focus group participants tended to perceive biological carbon removal as tangible, distributed, and permissive of proactive behaviour, allowing individuals to make sense of and engage with a systemic problem^17,18^. This applied to all approaches that relied upon biological sinks - but was especially strong for tree-planting. Indeed, the positive application of naturalism, feasibility, and agency to tree-planting was the only universal perspective identified in the study, represented in all 22 countries^17^.

Crucially, as we show in Table 1, participants often compared tree-planting favourably in relation to engineered carbon removals, such as Direct Air Capture, which were seen as costly, resource-intensive, and leveraging the same industrial processes that were perceived to be the root causes of climate change and unsustainability.

**Table 1:** Focus group quotes

Theme	Representative quote
Planting trees represents a complex of naturalism, feasibility, and agency, in comparison to engineered CDR	Turkey, Urban: I don’t think it’s necessary to capture and store carbon, relying on large-scale engineering and chemical systems for that. It is the easiest and most effective way and the most harmless and most beneficial way: planting a tree.
	Austria, Rural: … it changes nature back to the way it was before we started affecting it… I think it’s an ideal accompanying measure. And above all, it’s more manageable technologically (than direct air capture). So to every regular person, this seems less abstract than the other approaches.
	Brazil, Urban: I don’t see any risks, I see more benefits, these ideas (tree-planting) are taking advantage of natural resources, it’s about not having all these greenhouse gases, and chemical products (e.g. direct air capture), it’s something more natural. I think it’s worth investing in these.
	Kenya, Urban: K: Yes. High energy costs will mean that the petrol prices will go up higher than they already are. That means I will spend more than I spend. J: Actually, let’s compare it to planting trees then. Cheap, cheap but at the end of the day I will cut this tree, and compensate with another one here, right?
Non-biological CDR juxtaposed as costly, resource intensive, industrial, and reflecting root causes of unsustainable behaviour	Brazil, Rural: The other ideas are always connected to engineering, you have to spend money in order to work and it is a constant, you have to do it all the time, at the moment you stop doing it, the effect ceases. If you plant a tree, that’s it… if you removed one, plant another 50. It’s not that difficult to plant a tree, there’s a technique, but much less specific than having to store energy or something like that.
	Italy, Rural: We created the imbalance in nature… Obviously, when we speak about planting trees, we are trying to re-balance the situation. We are not doing anything different than what we initially found. Working on planting trees is something natural. It’s not natural, creating industries that have to manufacture systems that restore the balance. We are building more and more and that creates further pollution, temperature increases and so on.
Addressing “root causes”, by reversing elements of industrial civilization or the human footprint	Sweden, Rural: As I said before, there is much that we can blame ourselves about, and to stop cutting down rainforests, and to try to replant… Others are, as mentioned, a bit high tech, and not tested either. I don’t know. But to take care of what we have, and to try to recreate what we have destroyed ourselves.
	South Africa, Urban:… Trees take many years for it to grow to full fruition. So that’s more of a longer term approach. But I think we need to start thinking of long term solutions because it took us a long time to get to where we are. So we need to find solutions that will change habits instead of like shortcuts; because once you do short cuts people are just gonna continue living the way they are.
	Indonesia, Rural: It is actually, like, we’re back to nature. We’ve exploited the earth a lot. So, it should be, like, we’re back to earth. And since I have this faith, like, back to ‘fitrah’ (natural tendency), back to nature. We have to go back, no matter how, to replant trees. We have to… we shouldn’t keep causing damages. Like, those industries popping up everywhere.
Over-optimism about prospects of tree-planting	Saudi Arabia, Urban: Since the place we live is high in temperature and carbon emissions, so it can all be solved by using very simple methods, right now the afforestation plan is being done in Riyadh which will help in absorbing carbon and decreasing the heat, so this is amazing and it’s important as well as it affect the health, life, and food, it affects everything.
	India, Rural: Yeah, actually it is useful to all human beings and living life because the gardening of plants and trees can benefit all categories of people and who have good land. So mostly this can be done in towns because in those places we can see much agriculture and when I talk about cities here, we can see only big buildings, apartments, and complexes. So, there is no sufficient place for gardening. And you know this is cost less, so through planting the many plants we can get fresh oxygen and help to reduce carbon dioxide.

Representative quotes from 44 online focus groups in 22 countries, with one urban and one rural group in each country (n = 323). For additional data and supplementary methodological materials, see refs. ^17–19^.

Participants highlighted a host of co-benefits associated with tree-planting that spanned biodiversity, health and air quality, flood prevention and soil erosion^17^, often conditioned by experiences of climate or environmental risk^19^. Yet the benefits were sometimes over-stated, or assumed to be so systemic that support could border on the simplistic: for example, commonly stating that “everyone” would tangibly benefit from afforestation efforts. Participants did not intuitively grasp nuances between afforestation, reforestation, and avoided deforestation, and there was very little discussion of different kinds of trees, their permanence, differences in carbon sequestration and suitedness to different areas - all of which have difficult carbon accounting and policy implications.

We should be cautious about overstretching the interpretation of our data. Favouring tree-planting was not always one-dimensional or overly enthusiastic, especially in the focus groups. The sense that trees distract from other types of carbon removal was common - but not universal. Participants also recognized risks in tree-planting, including land-use trade-offs, climate impacts from non-permanent biological carbon stocks, questions over potential impermanence, and perverse profit motives.

Techniques such as Direct Air Capture also maintained a significant degree of public support, especially in countries with more faith in innovation and/or state-owned industry sectors (India, China, Saudi Arabia, Norway, Switzerland). Nevertheless, there is clear evidence that tree-planting takes up a disproportionate space in the public imagination of carbon removal.

## Conclusion

In sum, we suggest that the notion of tree planting as a carbon removal technique could act to bias public perceptions more negatively toward other carbon removal techniques. In our data, particularly our qualitative studies, we have witnessed this effect for a wide range of different carbon removal techniques, including geological, electrochemical, and biological methods, in locations around the world. Trees are perceived as natural, intuitive, providing co-benefits, and acting on a more preferable timescale in a context of climate urgency. In various projects, we attempted to balance this by communicating the huge scale of tree planting and land-use change which would be required, or by emphasising the need for permanent storage. None of this appears to have made a difference.

Essentially, trees have become tightly interwoven with notions of naturalness, and in particular with the Romanticist view of nature which sees nature as external, pure and pristine^20^. Nevertheless, nature as a concept is socially constructed and can be reconstructed in different ways. Future research and communications could seek new ways of challenging this view, to avoid detracting from other vital policy options. One option would be to emphasise the naturalness in all climate interventions to put them all on a level playing field^8^; another would be to emphasise the technology inherently involved in tree planting at climate-relevant scales, in order to defuse the benefits they receive from the nature framing^21^.

What is now needed is targeted research to empirically test the existence and extent of this bias. Experimental studies could test whether the presence of an option of tree planting indeed systematically reduces support for other approaches. Qualitative data and mixed methods will be particularly valuable for unpicking the underlying (and often complex) reasons behind participants’ responses and opinions for supporting planting trees, including the way that it is seen as a desire for agency in the face of a systemic problem.

We should also be wary of policy that mispurposes the general enthusiasm for tree-planting, such as policies which support carbon forestry involving poor-quality credits or land grabs, and those which promote the false equivalence of different kinds of removals^22^. Taking this one step further, perhaps we should query the way in which tree planting is often conflated with climate action, and instead focus tree-planting policies on its other benefits, such as biodiversity support, flood prevention, air quality improvement, and wellbeing.

Going forward, it will be crucial to understand trends in public opinion, and to identify emerging areas of contention or bias: these could have a significant impact on the prospects for scaling up promising innovative approaches.
